# Membrane-domain mutations in respiratory complex I impede catalysis but do not uncouple proton pumping from ubiquinone reduction

**DOI:** 10.1093/pnasnexus/pgac276

**Published:** 2022-12-02

**Authors:** Owen D Jarman, Judy Hirst

**Affiliations:** The Medical Research Council Mitochondrial Biology Unit, University of Cambridge, The Keith Peters Building, Cambridge Biomedical Campus, Hills Road, Cambridge CB2 0XY, UK; The Medical Research Council Mitochondrial Biology Unit, University of Cambridge, The Keith Peters Building, Cambridge Biomedical Campus, Hills Road, Cambridge CB2 0XY, UK

**Keywords:** biological energy transduction, electron transport chain, respiratory chain, NADH:ubiquinone oxidoreductase, proton pumping

## Abstract

Respiratory complex I [NADH:ubiquinone (UQ) oxidoreductase] captures the free energy released from NADH oxidation and UQ reduction to pump four protons across an energy-transducing membrane and power ATP synthesis. Mechanisms for long-range energy coupling in complex I have been proposed from structural data but not yet evaluated by robust biophysical and biochemical analyses. Here, we use the powerful bacterial model system *Paracoccus denitrificans* to investigate 14 mutations of key residues in the membrane-domain Nqo13/ND4 subunit, defining the rates and reversibility of catalysis and the number of protons pumped per NADH oxidized. We reveal new insights into the roles of highly conserved charged residues in lateral energy transduction, confirm the purely structural role of the Nqo12/ND5 transverse helix, and evaluate a proposed hydrated channel for proton uptake. Importantly, even when catalysis is compromised the enzyme remains strictly coupled (four protons are pumped per NADH oxidized), providing no evidence for escape cycles that circumvent blocked proton-pumping steps.

Significance Statement Respiratory complex I is a key player in metabolism, coupling NADH oxidation and ubiquinone reduction to proton transfer across an energy-transducing membrane to power ATP synthesis. However, despite substantial advances in structural knowledge on complex I, its mechanism of catalysis, including how and where protons are pumped across the membrane, remains obscure. Here, we use a powerful bacterial model system, *Paracoccus denitrificans*, to investigate 14 mutations of key residues and structural features in the membrane-domain Nqo13/ND4 subunit, one of three antiporter-like subunits, and evaluate their roles in proton translocation. Notably, we do not observe enzyme uncoupling. Our data advance understanding of the mechanism of catalysis and challenge previous reports of “escape cycles” that avoid blocked proton-pumping steps.

## Introduction

Respiratory complex I [NADH:ubiquinone (UQ) oxidoreductase] is a crucial metabolic enzyme central to oxidative phosphorylation (1, 2). By catalyzing NADH oxidation and UQ reduction, it regenerates NAD^+^ to sustain essential metabolic processes, including the tricarboxylic acid cycle and fatty acid oxidation. In addition, the energy from the oxidoreduction reaction is captured and coupled to translocate four protons (3) across the inner mitochondrial membrane, or cytoplasmic membrane in prokaryotes, contributing to the proton motive force (Δp) that drives ATP synthesis and transport processes. These vital metabolic roles of complex I, along with its capacity to produce reactive oxygen species (ROS) (4, 5), make complex I dysfunctions the origin for a wide range of mitochondrial diseases and disorders (6, 7).

**Fig. 1.**
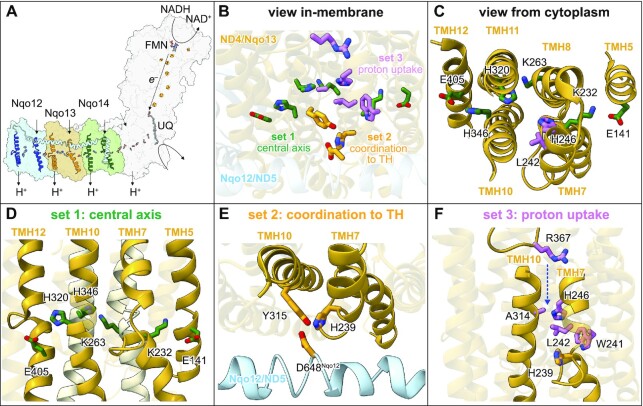
Overview of the residues mutated in *Paracoccus denitrificans* complex I. (A) Summary of the key mechanistic features in complex I. NADH is oxidized at the flavin mononucleotide (FMN), and electrons are transferred to UQ via a chain of iron-sulfur clusters. Key conserved charged residues that connect the UQ site to the proton pumping machinery and the transverse helix (TH), which spans the membrane domain, are shown. The three antiporter subunits are highlighted in blue (ND5/Nqo12), orange (ND4/Nqo13), and green (ND2/Nqo14), with their suggested sites of proton uptake and exit at discontinuous helices TMH5 and TMH12 in each subunit also shown. (B) Overview of the three groups of residues mutated in the ND4/Nqo13 subunit (and the TH of ND5/Nqo12) viewed from in the membrane: the central axis of conserved residues (set 1, green), coordination of the Nqo12-TH to Nqo13 (set 2, orange), and the proton uptake channel residues (set 3, pink). (C) The relative positions of the residues in the central axis of ND4/Nqo13 and the conserved residues H246 and L242 in the proposed proton-uptake/hydration channel, viewed from the cytoplasm. (D) The residues of the central axis in ND4/Nqo13. (E) Coordination of the TH to ND4/Nqo13 via D648^Nqo12^ on the TH and Y315 and H239 on TMH10 and TMH7 of ND4/Nqo13, respectively. (F) Residues involved in the proposed proton-uptake/hydration channel. The conserved Leu-His-Trp triad is shown along with an Ala on TMH10, a His on TMH7, and the R367 residue mutated in the R367H LHON variant. All images were created using the structure of bovine complex I (PDB: 7QSK) (35) in which all the residues shown are conserved, with residue numbers given for *P. denitrificans*. The residues mutated and their equivalent numbers in other common model organisms are summarized in Table S1.

The conserved catalytic core of complex I contains 14 subunits, seven in the hydrophilic domain and seven in the membrane domain, dedicated to electron transfer and proton pumping, respectively (8–10) (Figure 1A). However, despite a recent wealth of structural information, both the mechanism by which the redox energy is captured and coupled to drive proton pumping and the pathways for proton uptake and export remain unclear. Three homologous antiporter-like subunits (ND2/Nqo14, ND4/Nqo13, and ND5/Nqo12) have been proposed as three individual modules for proton uptake and ejection, with a putative fourth module located within the ND1/ND3/ND4L/ND6 (Nqo8/Nqo7/Nqo11/Nqo10) subunits (8, 11, 12). Conversely, it has also been suggested that all four protons are ejected from ND5/Nqo12, since the only exit channel readily identifiable in cryo-EM analyses with waters present is located there (10, 13, 14). Clearly, substantial questions remain to be answered on the roles of each antiporter-like subunit in proton pumping and complex I catalysis.

Both structural data and computational modeling have identified residues in the membrane domain that are likely key to catalysis (9, 10, 12, 13, 15, 16). However, direct investigations to confirm their roles, by site-directed mutagenesis combined with in-depth biophysical and biochemical analyses, are currently lacking. Many mutations have been created in complex I from *Escherichia coli* (17–23), but most studies predated detailed structural knowledge and relied only on rates of NADH oxidation, thereby lacking information on energy conversion and proton-pumping stoichiometries. Recently, we developed the α-proteobacterium *P. denitrificans* as a versatile model system for mitochondrial complex I that enables comprehensive studies of membrane-domain mutations in a robust, well-characterized system (24) demonstrated previously for quantitative determination of proton-pumping stoichiometries and measurements of reverse catalysis [reverse electron transfer (RET) through Δp-linked ubiquinol:NAD^+^ oxidoreduction] (3, 25, 26). Here, using this model system, we investigate three aspects of catalysis by the antiporter-like subunits by site-directed mutagenesis of the ND4/Nqo13 subunit (Figure 1A to C).

First, a chain of conserved charged residues along the “central axis” of the membrane domain connects the UQ-binding site and the proton-pumping modules (8, 9, 15) (Figure 1B to D). A common motif, conserved in each antiporter-like subunit, includes a Glu-Lys ion pair between TMH5 and TMH7 as the axis enters the subunit (from the UQ-binding site) and a terminal Lys or Glu on TMH12 as it exits it or ends. These residues have been proposed to undergo protonation/deprotonation and conformational changes during turnover and to respond to changes in hydration (12, 27). Here, we confirm that charged residues along the central axis are essential for catalysis and show that mutations of them that impede catalysis impede both NADH:UQ oxidoreduction and proton pumping equally: they do not create catalytic “escape” cycles that avoid blocked proton transfer steps and exhibit decreased proton-pumping stoichiometries.

Second, a transverse helix (TH) in the C-terminal domain of subunit ND5/Nqo12 runs from the tip of the membrane domain along the sides of subunits ND5/Nqo12, ND4/Nqo13 and ND2/Nqo14, appearing to strap them together (Figure 1A and E). Some studies have advocated it only has a structural role (28, 29), but others have proposed it acts as a piston or “coupling rod” to synchronize the proton-pumping modules (8, 30, 31) and mutational studies have reported decreased proton-pumping stoichiometries when the ND4/Nqo13 subunit is disconnected from the TH or the TH is truncated (32, 33). Here, we used mutations to disconnect the TH from ND4/Nqo13, producing enzymes with decreased stability but the same proton-pumping stoichiometry as the wild-type. Therefore, the TH fulfils a purely structural role.

Finally, proton uptake by the antiporter-like subunits is thought to involve a hydrated channel between TMHs 7b, 8 and 10 that connects the mitochondrial matrix/bacterial cytoplasm (negative, N-side of the membrane) to the central axis (12, 16) (Figure 1C and F). Here, mutations of highly conserved residues in and around the channel were generated to disrupt proton uptake and/or hydration, or to permanently open or close the channel and decouple proton transfer from redox catalysis. The results support the location of the proton-uptake channel and highlight individual residues important for catalysis. However, all the variants remained tightly coupled and, again, exhibited only the same proton-pumping stoichiometry as the wild-type enzyme.

Our data provide new information on the roles of key residues during turnover and a basis for evaluating candidate models for the mechanism of complex I catalysis. In particular, in every variant NADH:UQ oxidoreduction is tightly coupled to proton pumping, such that neither reaction can occur without the other, and the proton-pumping stoichiometry is strictly conserved, even when catalysis is substantially impeded.

## Results

### Design and creation of specific site-directed mutations

The conserved Glu and Lys residues that span the ND4/Nqo13 subunit on the central axis (Figure 1B to D) were mutated to Gln to remove their charges while preserving their polarity and size. The E141Q and K232Q mutations target the ion-pair where the axis enters the subunit, the K263Q mutation (24) targets a Lys residue on TMH8 conserved in ND4/Nqo13 and ND2/Nqo14, and E405Q targets the terminal Glu. [Note that all residue numbers refer to *P. denitrificans* complex I, see Table S1 for the equivalent numbers in other common model organisms]. In ND4/Nqo13, a pair of conserved His residues (H320 and H346) are also present on the central axis (Figure 1B to D). As they probably have similar roles to each other, one was mutated to Gln (H346Q) to prevent proton exchange but maintain polarity, and the other to Leu (H320L) to introduce a nonpolar aliphatic side chain. The connection between the carboxylate-residue D648^Nqo12^ on the TH and H239 and Y315 in ND4/Nqo13 (Figure 1E) was destabilized by creating the D648N^Nqo12^, H239Q, and Y315F variants. The precise nature of this connection is unclear (direct hydrogen or ionic bonding or coordination via bridging waters), as structures from different species have exhibited a range of D648^Nqo12^ to H239/Y315 distances (2.4 to 4.6  and 2.5 to 4.3 Å, respectively) (34, 35). In the proposed proton-uptake/hydration channel (Figure 1F), the conserved Leu-His-Trp triad, observed in cryo-EM data on *Yarrowia lipolytica* complex I to be restructured in the more-hydrated ND2/Nqo14 subunit relative to in the less-hydrated ND5/Nqo12 and ND4/Nqo13 subunits (15), was targeted by the L242A, H239Q, and W241F mutations. In particular, the Leu sidechain, suggested to move in and out of the channel during turnover, was replaced by the shorter Ala sidechain to prevent it closing the channel effectively. Moving up the channel, the conserved Ala (TMH10) that protrudes into the channel and contacts L242 was mutated to Leu (A314L) to attempt to block the channel, and the H246F variant (TMH7) was created to assess the role of the His in proton transfer and channel hydration (10, 12). Finally, at the top of the channel, R367 has been observed to coordinate a water molecule (15). The R367H mutation is an established cause of Leber's hereditary optic neuropathy (LHON) (36, 37), which leads to vision loss in patients, suggesting an important functional role for the residue. The R367H mutant was created to investigate this possibility.

The point mutations in Nqo13 and Nqo12 were generated in a strain of *P. denitrificans* developed for studying complex I. Its genome has been modified to encode a tag for enzyme purification, and the alternative NADH dehydrogenase (NDH-2) from *E. coli* has been introduced on an expression vector to allow the study of inactive complex I variants (24). The mutations were created as unmarked chromosomal substitutions by suicide vector-mediated homologous recombination, as described previously (3, 24, 38) and verified by sequencing.

### Catalytic activity of complex I variants

**Fig. 2.**
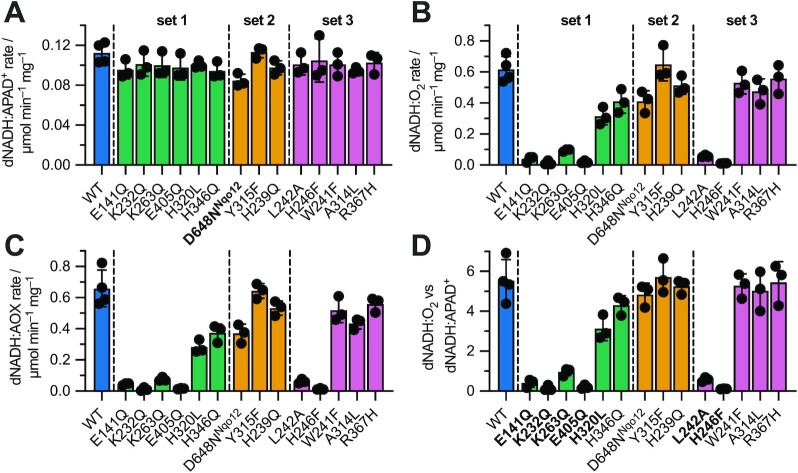
Catalytic activities for complex I variants measured in membranes. Variants are organized into the three sets defined in Figure 1D to F. At least three biological replicates were prepared for each variant and shown as individual points, with the bar giving the mean average and the error bar the standard deviation. (A) dNADH:APAD^+^ oxidoreduction by the complex I flavin site. Statistical significance was calculated by a one-way ANOVA and Dunnett's test comparing all variants to wild type. Only D648N^Nqo12^ (bold) differed significantly from the wild type (*P* < 0.05). (B) dNADH:O_2_ oxidoreduction by complexes I, III, and IV following subtraction of piericidin A-insensitive background rates. (C) dNADH:AOX activity: dNADH:O_2_ oxidoreduction by complex I and AOX following subtraction of piericidin-insensitive background rates. Membranes were supplemented with 20 µg mL^–1^ AOX and complexes III and IV inhibited with 1 mM antimycin A and 400 µM KCN, respectively. (D) The dNADH:O_2_ activity is normalized by the amount of complex I present, represented by the dNADH:APAD^+^ activity. Each point represents the dNADH:O_2_/dNADH:APAD^+^ ratio from an individual preparation. Statistical significance was calculated by a one-way ANOVA and Dunnett's test comparing all variants to wild type with variants that differed significantly from wild type labeled in bold (*P* < 0.0001 for E141Q, K232Q, K263Q, E405Q, L242A, and H246F, and *P* < 0.001 for H320L).

Figure 2 shows the catalytic activities of each variant measured in *P. denitrificans* membranes. Three biological replicates were prepared for each variant from individual cell cultures, with cell growth supported by NDH-2 expression when cells either did not grow without it or achieved much lower cell densities in overnight cultures than the wild type (the E141Q, K232Q, K263Q, E405Q, H320L, L242A, and H246F variants). The D648N^Nqo12^ variant was cultured to sufficient yields without NDH-2 expression, although higher cell densities could be achieved by inducing it. First, the amount of complex I present in each preparation was assessed using an assay specific to the complex I flavin site (deaminoNADH:APAD^+^ [dNADH:APAD^+^] oxidoreduction), which is distant from the membrane and assumed unaffected by the status of the proton-pumping subunits (Figure 2A). Only the value for the D648N^Nqo12^ variant was decreased significantly relative to the wild type, suggesting an assembly or stability defect. Then, NADH:UQ catalysis was measured for each variant as the dNADH:O_2_ activity, using the fact that dNADH is a complex I-specific substrate (39), and complexes III and IV to reoxidize the ubiquinol and link NADH oxidation to O_2_ reduction (Figure 2B). NADH:UQ catalysis was also investigated using the alternative ubiquinol oxidase (AOX) from *Trypanosoma brucei brucei* instead of complexes III and IV to reoxidize the ubiquinol and reduce O_2_ (Figure 2C) (40). The results of the CI:CIII:CIV and CI:AOX assays (Figures   2B and C) show remarkable consistency, confirming that the effects observed are due to complex I catalysis and not downstream components. Finally, to compare NADH:UQ activities between variants independently of differences in complex I content between preparations, the data in Figure   2B were normalized using the data in Figure 2A. The results (Figure 2D) show that NADH:UQ oxidoreduction is severely diminished for variants E141Q, K232Q, K263Q, E405Q, L242A, and H246F. H320L showed an intermediate decrease in catalysis (57% of the wild-type value) that is statistically significant. All other variants (H346Q, D648N^Nqo12^, Y315F, H239Q, W241F, A314L, and R367H) exhibited activities that were not significantly different from the wild-type activity.

### Assembly and structural integrity of complex I variants

**Fig. 3.**
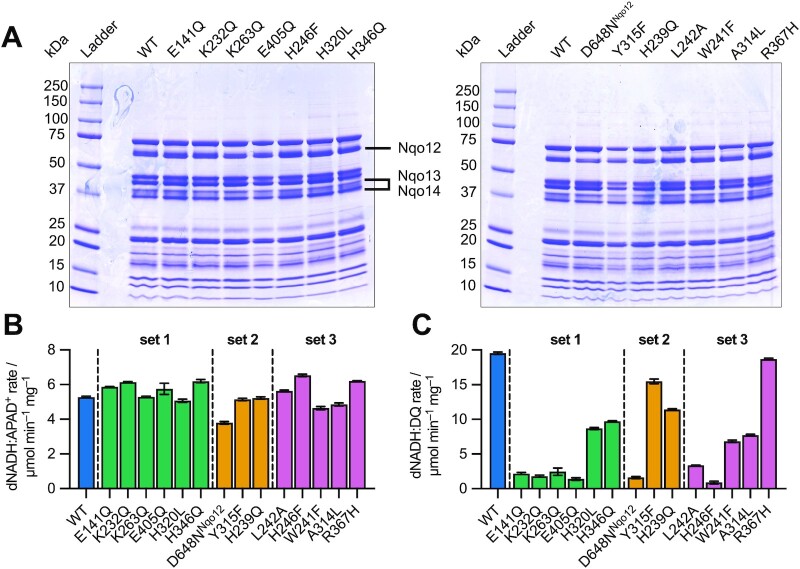
Characterization of purified wild type and variant complexes I. (A) SDS-PAGE analyses of the purified enzymes showing the conserved banding pattern. The bands for the three antiporter-like subunits are marked, based on mass spectrometry analyses of the wild-type enzyme (24). (B) The dNADH:APAD^+^ activity of each purified variant. (C) The dNADH:DQ activity of each purified variant, following subtraction of piericidin A-insensitive background rates. The activities for each variant were measured in technical triplicate and shown with SEM values. Statistical significance was assessed by one-way ANOVA and Dunnett's test comparing each variant to the wild type. All variants recorded a *P* value < 0.0001, apart from R367H, which was not significant. For reference, Figure S4 shows the activities of the specific membrane preparations used to purify the variants.

To investigate their structural integrity and protein composition, each variant was purified (24) and its elution profile in size-exclusion chromatography was compared to that of the wild-type enzyme (Figure S1). Nearly all variants exhibited elution profiles and volumes (1.17 mL) essentially identical to the wild-type, indicating the complexes were fully formed and intact. Only the D648N^Nqo12^ variant showed a slight delay in elution (1.22 mL), suggestive of a structural perturbation or loss of stability. In SDS-PAGE analyses, all the variants, including D648N^Nqo12^, were essentially indistinguishable from wild-type, so they all contain the expected complement of subunits (Figure 3A). There was no obvious evidence of NDH-2 contamination (expected at ∼47 kDa) in the purified samples, even when NDH-2 was expressed for cell growth.

To further assess the complex I stabilities, nanoscale differential scanning fluorimetry (nanoDSF) was performed on the isolated complexes (Figure S2). The fluorescence signal is monitored as the sample temperature is gradually increased, and the data are presented as the first derivative of the signal intensity. Two peaks were observed in all cases. The second peak (66.3 ± 0.1 °C for wild-type) matches the temperature (67 to 68 °C) at which the FMN dissociates from *E. coli* complex I (41) suggesting that it represents unfolding of the hydrophilic domain. The first peak (44.6 ± 0.2 °C for wild-type) thus probably represents unfolding of the detergent-solubilized membrane domain. The profiles from variants E141Q, E405Q, H320L, and R367H were nearly identical to wild-type, while the other variants showed a range of changes to the peak shapes and temperatures, suggesting localized changes in stability of the membrane domain that propagate out. In particular, the purified D648N^Nqo12^ variant appears markedly destabilized, with the first peak sharpened and both peaks shifted to lower temperature (Figure S2). The Y315F and H239Q variants (mutations of residues that interact with D648^Nqo12^) were less severely affected.

The dNADH:APAD^+^ specific activities for the purified variants (Figure 3B), reflecting the integrity of their flavin sites as well as their purity, are all comparable, except the activity of the D648N^Nqo12^ variant is relatively low, consistent with its decreased stability. In agreement with the data from the membrane assays, the purified E141Q, K232Q, K263Q, E405Q, L242A, and H246F variants were unable to catalyze NADH:UQ oxidoreduction. Strikingly, NADH:UQ oxidoreduction by purified D648N^Nqo12^ was also abolished, despite its near-wild-type value in membranes. Insertion of the D648N^Nqo12^ enzyme into proteoliposomes did not rescue the activity, despite the removal of DDM (the detergent *n*-dodecyl β-D-maltoside) and reconstitution of a lipid membrane around the protein (Figure S3). The loss of activity is consistent with the significant decrease in stability of the purified D648N^Nqo12^ enzyme revealed by nanoDSF. Otherwise, while the R367H mutant retained wild-type behavior following purification, the remaining variants (H320L, H346Q, Y315F, H329Q, W241F, and A314L) exhibited intermediate activities (35% to 79% of wild-type). The activities of W241F and A314L, in particular, decreased in the isolated enzymes relative to in membranes, suggesting that, like D648N^Nqo12^, they also lose activity upon solubilization in DDM.

### All catalytically active complex I variants pump four protons

To assess whether proton pumping in one or more antiporter-like subunits is disrupted by any of the mutations in ND4/Nqo13, the number of protons translocated per NADH oxidized (the proton-pumping stoichiometry, *n*^CI^) was determined for each catalytically competent variant using a protocol modified from that established previously for bovine submitochondrial particles (SMPs) and *P. denitrificans* sub-bacterial particles (SBPs) (3, 40). Briefly, *n*^CI^ is calculated by comparing the rates of oxidoreduction and ATP synthesis during catalysis by different respiratory pathways that pump different numbers of protons per turnover. SMPs and SBPs are well-coupled, inverted vesicles of native membranes. Here, the three pathways used in *P. denitrificans* SBPs were catalyzed by complexes I, III, and IV (CI/CIII/CIV, (*n*^CI^ + 6) H^+^ per NADH oxidized), complexes II, III, and IV (CII/CIII/CIV, 6 H^+^ per succinate) and complex I plus AOX (CI/AOX, *n*^CI^ H^+^ per NADH), where the 2*e*^–^ proton-pumping stoichiometries of CIII and CIV are well-established (42, 43) (and CII and AOX do not pump protons). The CI/AOX pathway was created by addition of exogenous AOX to the SBPs, with 1 mg AOX per mg SBPs chosen to secure a substantial rate of substrate oxidation while minimizing losses of efficiency observed at saturating concentrations. AOX was added to all experiments for consistency, and catalysis by AOX or CIII/CIV was enforced by addition of 1 µM antimycin A and 400 µM KCN to inhibit CIII/CIV or 1 µM ascofuranone to inhibit AOX, respectively.

**Fig. 4.**
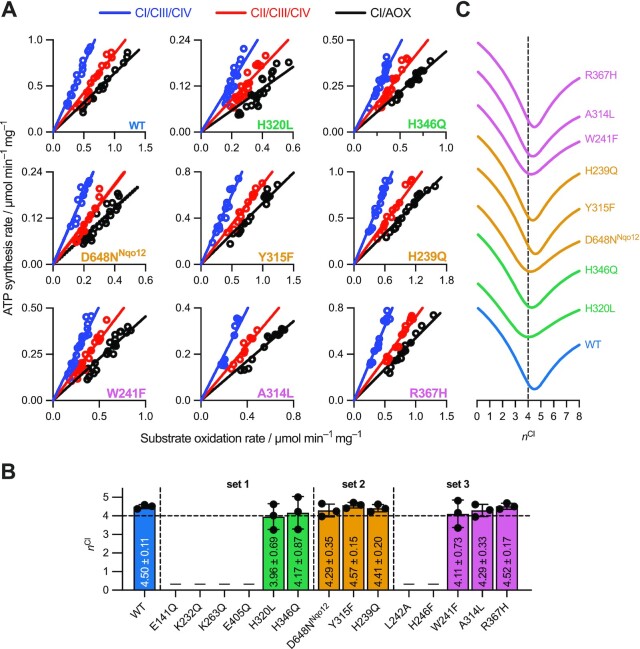
Determination of the proton-pumping stoichiometry for each complex I variant in SBPs. (A) ATP synthesis rates for three pathways are shown over a range of substrate oxidation rates: CI/CIII/CIV (blue), CII/CIII/CIV (red), and CI/AOX (black). Oxidation rates of CI- and CII-driven pathways were modulated by titration of inhibitors piericidin A and atpenin A5, respectively. Individual lines of best fit, passing through the origin, were fit to the raw data for each pathway. (B) Calculation of *n*^CI^ for each variant based on pairwise comparisons of the best-fit gradients for each pathway, which are shown as individual points on the bar chart (average *n*^CI^ value) with the SD. (C) Global fitting of the wild-type and variant datasets to simulated *n*^CI^ values, which fix the relative gradients of the pathways through Equations 1**–**3. The curves show the sum of the squared residuals for the fits at each *n*^CI^ value; the dotted line is at *n*^CI^ = 4.

Overlapping ranges of ATP synthesis rates were generated for each pathway by the titration of complex I and II inhibitors (piericidin A and atpenin A5, respectively), and plotted against the corresponding rates of substrate oxidation (Figure 4A). Fitting the data empirically using lines that pass through the origin then allows us to compare ATP synthesis rates driven by different substrate oxidation pathways over a wider kinetic range than in previous single-point comparisons (3), on the assumption that the rate of proton leak is the same for each pathway when the rate of ATP synthesis (and therefore Δp) is the same. First, pairwise comparisons of the gradients of the best fit lines for each pathway were used to derive three values for *n*^CI^ (Figure 4B). These values and their averages indicate that every variant, like wild type, pumps four protons per NADH oxidized. Second, a given value of *n*^CI^ sets the relationships between the three gradients, allowing the best fit to the data for that value to be calculated by minimizing the sum of the squares of the differences between the data and the fit (the error of the fit, see the "Materials and Methods" for details). Figure 4C shows how the error of the fit depends on *n*^CI^ for each variant, with clear minima between 4.0 and 4.6 in all cases. It is currently unclear why the values tend to drift above 4 (as is also evident in Figure 4B) however this small, systematic error is conserved in both the wild-type and across the variants (one possibility is low-level expression of alternative ubiquinol oxidases). Finally, our data are compared to the simulated best fits for *n*^CI^ = 2, 3, and 4 in Figure S5, where the fits from *n*^CI^ = 2 and 3 are clearly unsatisfactory whereas *n*^CI^ = 4 predicts convincing fits to the datasets. Our analyses clearly show that no variant exhibits a decreased proton-pumping stoichiometry of less than four protons per NADH oxidized.

### All catalytically competent variants catalyze RET

**Fig. 5.**
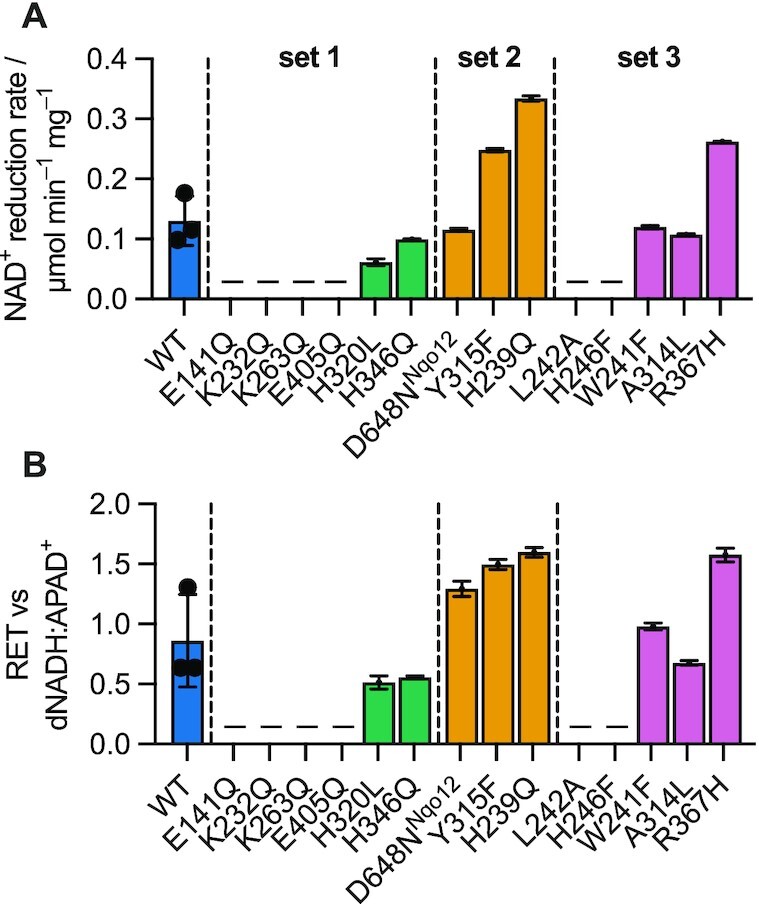
RET catalysis by *P. denitrificans* SBPs with variant and wild-type complex I. (A) The NAD^+^-reductase activity in SBPs was measured for mutants that possessed significant NADH oxidation activity in membranes. Variants not included are marked by a dash. All measurements are the average of three technical replicates ± SD. Three independent SBP preparations for the wild type were prepared and assayed to provide biological replicates to indicate the likely spread present in all data between SBP preparations (shown as individual points). (B) NAD^+^ reduction rates (RET) normalized to the dNADH:APAD^+^ activities. None of the variants differed significantly from wild type as calculated by one-way ANOVA and Dunnett's test.

The capacity of each complex I variant to catalyze NAD^+^ reduction in the RET (Δp-linked ubiquinol:NAD^+^ oxidoreduction) reaction was assessed. Based on published protocols (25, 26), RET in SBPs was driven by succinate oxidation to reduce the UQ-pool and complex III and IV catalysis to generate Δp. Rates of RET were measured as initial rates of NAD^+^ reduction (Figure S6), and the rate from wild-type SBPs [0.13 ± 0.02 µmol min^–1^ (mg SBP)^–1^] was found to be comparable to rates determined for bovine SMPs [0.05 to 0.13 µmol min^–1^ (mg SMP)^–1^] (40, 44, 45). Differences in the rates of RET were seen between the variants measured (those that are catalytically competent for NADH:UQ oxidoreduction), however, the spread in data between biological replicates of wild-type SBPs suggests the differences should not be overinterpreted (Figure   5). Although the “raw” rates in Figure 5A can be corrected for differences in complex I content between preparations using the data from dNADH:APAD^+^ assays (Figure 5B), we have not attempted to correct for differences in succinate oxidation or in how well-coupled (proton-leaky) the SBP membranes are. Nevertheless, our data demonstrate clearly that every variant capable of NADH:UQ catalysis is also capable of RET, consistent with a proton-pumping stoichiometry of (at least) four in every case. In RET, a substantial Δp is required to overcome the substantial (two-electron) reduction potential difference between ubiquinol and NAD^+^, and in the case of a variant, which pumps fewer than four protons, an even larger Δp would be required to overcome this redox gap. The ability to catalyze RET also confirms the tight coupling of electron transfer and proton transfer in all cases.

## Discussion

### Mutations in Nqo13/ND4 do not uncouple proton pumping from redox catalysis

The 14 variants of the*P. denitrificans* complex I studied here display a wide range of catalytic rates. Some are essentially indistinguishable from the wild-type, some are virtually inactive, and some exhibit intermediate rates (Figure 2). However, none of the functional variants display an altered proton-pumping stoichiometry. Their conserved values of four protons pumped for each NADH oxidized were determined directly using robust measurements combining data from three respiratory pathways (Figure 4), and are consistent with observations that they can also all catalyze RET (which requires a substantial input of energy from Δp) (Figure 5). Thus, when a mutation in Nqo13/ND4 (or linked to it on the TH) slows down a reaction step, the whole catalytic cycle, including NADH oxidation, and UQ reduction, slows down with it. The mutations do not create catalytic escape cycles that avoid blocked proton transfer steps and exhibit decreased proton-pumping stoichiometries.

Intriguingly, several mutations in *E. coli* complex I have been reported to decrease the proton-pumping stoichiometry. First, the D648N^Nqo12^ variant was reported to pump three protons per NADH oxidized (32). However, the NADH:UQ oxidoreductase activities of both the wild-type and variant enzymes following reconstitution into proteoliposomes were extremely low (∼1.6 μmol min^–1^ mg^–1^, compared to 35.0 ± 0.2 μmol min^–1^ mg^–1^ for *P. denitrificans* complex I (Figure S3), and 32.8 μmol min^–1^ mg^–1^ reported recently for wild-type *E. coli* complex I in detergent (46)). Furthermore, the decreased stoichiometry was determined by single-point comparisons of ACMA (9-amino-6-chloro-2-methoxyacridine) fluorescence quenching (which provides an indirect and semiquantitative measure of ΔpH across a liposomal membrane). Second, variants of the two highly conserved H320 and H346 residues in *E. coli* ND4/Nqo13 were reported to exhibit decreased proton pumping stoichiometries (27). However, the activities of the enzymes reconstituted into proteoliposomes were not reported, and the proton stoichiometries were evaluated by single-point comparisons of ACMA fluorescence and Oxonol-VI absorbance (which provides an indirect and semiquantitative measure of Δψ across a liposomal membrane). Here, we show unambiguously that mutations of all three residues (D648N^Nqo12^, H320L, and H346Q) pump four protons per NADH oxidized, and we therefore challenge the conclusions of decreased stoichiometries for the matching *E. coli* variants (32, 27). Recently, a decreased proton stoichiometry for the E405K variant in *E. coli* ND4/Nqo13 has also been proposed on the basis of ACMA measurements on enzymes catalyzing at extremely low rates (∼0.2 μmol min^–1^ mg^–1^) in proteoliposomes (46). Although we have not studied the E405K variant here, we further question the validity of this conclusion, based on such low rates of turnover. We propose that, so far, no mutation created in subunit ND4/Nqo13 has disrupted the tight coupling between the redox reaction and proton pumping that maintains a strict stoichiometry of four protons pumped per NADH oxidized, to enable efficient and reversible catalysis by complex I. Determining whether it is possible to ever create uncoupling or reduced-stoichiometry mutations in complex I will require robust methods for defining the proton-pumping stoichiometry, such as that described here, and further investigation of functionally relevant residues throughout the enzyme. We note that uncoupling mutations have been identified in the redox-coupled proton-pump cytochrome *c* oxidase (respiratory complex IV) (47, 48), highlighting the possibility that similar uncoupling mutations in complex I will be found and will provide insights into the mechanism of catalysis in future.

### Lateral energy transduction through Nqo13/ND4

Coupling mechanisms have been proposed that propagate the energy from UQ reduction along the central axis of the membrane domain by electrostatic changes linked to protonation and deprotonation events. Based on computational simulations, Kaila and coworkers (27) proposed a single electrostatic pulse travels forward then back through the membrane domain in each cycle. In ND4/Nqo13, the forward pulse opens the Glu-Lys ion pair, dependent on subunit hydration, and TMH8-Lys protonation state, driving proton transfer from the TMH8-Lys to the terminal Glu. In the backward pulse, a proton is taken up from the N-side to the TMH8-Lys, the proton on the terminal Glu is ejected to the P-side, and the ion pair is reset. In a related mechanism, Kampjut and Sazanov (10) proposed two forward electrostatic waves, governed by protonation and deprotonation of two glutamates in ND4L/Nqo11. In this mechanism, ND4/Nqo13 pumps on the alternate wave to ND2/Nqo14 and ND5/Nqo12, and the Glu-Lys pair forms a switch that controls proton transfer from the TMH8-Lys to the terminal Glu. Our results confirm, like those of earlier studies (49, 50), that the Glu-Lys ion pair and terminal protonatable residue (E141, K232, and E405 in Nqo13/ND4) are essential for catalysis, consistent with both mechanisms. Recent data showing that the *E. coli* E405K variant retains ∼80% NADH:UQ oxidoreductase activity suggests flexibility in the identity and charge of the terminal residue, with both the ND4-Glu and ND2/ND5-Lys catalyzing effectively in this position in ND4/Nqo13 ( 46). A substantial decrease in NADH:UQ oxidoreductase activity was also observed here for the K263Q variant (Figure   2), supporting its central role, although the remaining activity (18% of wild type) was not sufficient for stoichiometry measurements. The *E. coli* K263A variant showed even higher rates of NADH:UQ oxidoreduction (35% to 55% of wild type) and retained proton-pumping activity ( 49, 50). These results suggest the specific chemical properties of the Lys are not critical and it may be replaced by a network of water molecules capable of supplying a proton to the terminal protonatable residue. We note that ND5/Nqo12 contains both the ion pair and a terminal protonatable residue, but does not contain a TMH8-Lys residue. Finally, Kampjut and Sazanov also proposed that the four pumped protons may be “redistributed” along the central axis to all be ejected from ND5 (10, 14). If this is correct, the terminal charged residue in ND4/Nqo13 would deliver protons to subunit ND5/Nqo12 as protons shuttle along the membrane domain.

Our mutations of the two highly conserved His residues (H320L and H346Q) that link the TMH8-Lys (K263) to the terminal Glu (E405) in Nqo13/ND4 show decreased NADH:UQ oxidoreduction (∼57% and ∼78%, respectively, of the wild-type activity in membranes, Figure   2), similar to mutations of the equivalent residues to Ala in *E. coli* (27). However, the data from *E. coli* complex I were interpreted (27) to suggest that they perturb the electrostatic pulse, disrupting proton pumping by ND4/Nqo13 and ND5/Nqo12 but allowing it to continue unhindered in the two upstream modules, decreasing the proton-pumping stoichiometry. Here, we clearly demonstrate this interpretation is not correct: both variants pump four protons per NADH oxidized and are able to catalyze RET. Snapshots of the proton transfer chain in molecular dynamics simulations suggest that even the double variant is able to use water molecules instead of the His residues (27) to establish a hydrogen-bonding network connecting K263 to E405. We conclude this ad-hoc water network is sufficient for energy transduction in the H320L and H346Q variants, although configuring it may slow proton transfer, relative to through the well-established His-network in the wild type. We note that different residues/networks are provided for proton transfer to the terminal residues in Nqo14/ND2 and Nqo12/ND5, consistent with this apparent flexibility.

### The TH of Nqo12/ND5 does not couple proton pumping

Neither of the two electrostatic pulse mechanisms discussed above (27, 10) include a role for the Nqo12/ND5-TH in coupling or coordinating proton pumping through the antiporter-like subunits. Studies in *E. coli* that have aimed to evaluate this suggestion by truncating, constraining, or otherwise altering the TH, or by disconnecting it from ND4/Nqo13, have reached different conclusions. Two studies proposed the TH fulfils only a structural and stabilizing role, perhaps clamping the three antiporter-like subunits together (28, 29). In two other studies, variants lacking the TH or with the D648N^Nqo12^ mutation (32, 33) were reported to exhibit decreased proton-pumping stoichiometries. Here, we show conclusively (Figures 4 and 5) that removing the connection between D648^Nqo12^ and Nqo13/ND4 (the D648N^Nqo12^, H239Q, and Y315F variants) does not decrease the proton-pumping stoichiometry. We therefore confirm that the TH does not couple or coordinate proton pumping through the antiporter-like subunits. On the other hand, all three variants (particularly D648N^Nqo12^) displayed decreased stability and loss of activity upon purification, and we suggest this results from DDM (detergent) molecules intercalating between the TH and Nqo13/ND4, leading to irreversible degradation when the TH-Nqo13/ND4 connection is already weakened. A DDM molecule has been observed between the TH and ND4 in bovine complex I (35), and simulations of mouse complex I in a lipid membrane showed a POPE (1-palmitoyl-2-oleoyl-sn-glycero-3-phosphoethanolamine) there, coordinating to D648^Nqo12^ and H239 (51).

### Proton uptake and hydration from the N-side to the central axis

Based on molecular dynamics hydration simulations and resolved waters in cryo-EM structures, TMHs 7b, 8, and 10 of the antiporter-like subunits have been proposed to form hydrated channels for proton import to the TMH8-Lys and central axis. Proton transfers have been suggested to be gated by water molecules, with the TMH8-Lys protonation state controlling the hydration, and hydration required to open the Glu-Lys ion-pair (12, 16). Movement of the conserved Leu in the Leu-Trp-His triad, gated by a Leu–His backbone hydrogen bond, has also been suggested to control hydration, opening and closing the channel during catalysis (24). Strikingly, the conservative L242A mutation essentially abolished catalysis, so replacing the Leu sidechain with the shorter Ala may prevent channel cycling, regardless of the TMH8-Lys protonation state (12, 16). However, the H239Q (His239 forms the hydrogen bond), W241F (W241 moves along with L242), and A314L (intended to block the channel) mutations did not significantly affect catalysis, and their proton-pumping stoichiometries were unchanged. In the MrpD (Nqo13) subunit of the complex I-related Mrp (multiple resistance and pH) complex, the W241A variant (W228A) also showed near-wild-type (86%) activity (52). Higher up in the channel and closer to the N-side, TMH11-Phe354 has also been suggested to control channel hydration in ND4/Nqo13 (13) through a gating mechanism similar to L242. It was not tested here, but mutating the equivalent residue in the Mrp complex (F341A) exhibited only a moderate effect (64% of wild-type activity) (53, 54). Across the channel from Phe354, the highly conserved TMH7-His246 has been proposed to be crucial for proton uptake (10, 12) and mutating it to a nonpolar Phe abolished catalysis. The His may either provide a proton “stepping stone” or support a water network (so the H246F mutation creates a hydrophobic block). Together, the L242A and H246F variants support proton uptake through the channel between TMHs 7b, 8, and 10. Future structural characterization of catalysis-blocking variants will be required to further define the interlinked proton-transfer and hydration processes that are required for proton uptake to the central axis during complex I catalysis.

In *P. denitrificans* complex I, the ND4/Nqo13 R367H variant is essentially indistinguishable from wild type, despite the ND4-R367H mutation in human complex I causing LHON (36). R367H is a conservative mutation at the entrance to the proposed proton-uptake channel, where the His appears (at least in *P. denitrificans*) sufficient for catalysis. We note that Arg367 is substituted by a Leu in *T. thermophilus* complex I (Table S1). The human ND4-R367H variant has been proposed to increase ROS production (37), to alter quinone substrate affinities, and to be less sensitive to rotenone inhibition (55). However, R367 is distant from both the flavin site of ROS production (56) and the UQ/rotenone binding site. Although an unexpected additional rotenone-binding site has been observed in ovine complex I, stabilized by ND4-R159, W241, and K232 (10), *P. denitrificans* complex I is less sensitive to rotenone than mammalian complex I (24) so is not a relevant model to investigate this site. It is simply likely that small differences in the *P. denitrificans* enzyme result in it failing to recapitulate the subtle pathogenic effects of the human mutation.

### Deconvoluting the pathways for proton translocation

Recently, it has been suggested that, instead of the four protons being pumped through four distinct pathways in complex I, all four protons are ejected from Nqo12/ND5 (10, 13, 14). Here, we have concluded that blocking individual proton-transfer sites or steps in an individual antiporter-like subunit blocks the whole catalytic reaction and therefore predict that blocking proton ejection from Nqo12/ND5 will similarly block the whole catalytic reaction, regardless of whether one or four protons are directly affected. While designing single-point mutants to distinguish the two proposals will thus be difficult, deleting one or more of the antiporter-like subunits may provide a way forward. Although deleting ND4/Nqo13 and ND5/Nqo12 in *E. coli* has not resulted in active enzymes, a serendipitous supernumerary subunit knockout in *Y. lipolytica* complex I generated a partially assembled enzyme that lacked ND5/Nqo12 and ND4/Nqo13, but otherwise retained its integrity. This enzyme displayed ∼38% of the wild-type NADH:UQ activity, and was suggested to pump two protons per NADH on the basis of the relationships between ACMA fluorescence quenching and NADH:UQ oxidoreduction rates in proteoliposomes (57). Recapitulating this experiment in *P. denitrificans* to confirm the decreased proton-pumping stoichiometry without reliance on ACMA quenching and enzyme reconstitution is now desirable. When antiporter-like subunits are missing, the single forward pulse proposed by Kaila and coworkers would need to be “reflected back” early (by ND2/Nqo14 not ND5/Nqo12 (27)) or the two pulses proposed by Kampjut and Sazanov would simply end early (10). Here we draw the following analogy: a blocking mutation acts like a cog that refuses to rotate, thereby preventing all the other cogs from turning, whereas a missing subunit, like a missing cog, offers no resistance. Further investigations using the *P. denitrificans* model system will be invaluable for investigating specific mechanistic questions such as these by allowing precision mutagenesis in the membrane-bound proton-transporting subunits to be combined with robust and quantitative evaluation of the kinetics and energetics of the coupled redox and proton-pumping reactions at the heart of this intriguing energy-transducing enzyme.

## Materials and methods

### Creation of complex I variants

Complex I variants were created in the *Pd*-Nqo5^His6^ strain described previously (24), which also contained the *E. coli ndh2* gene on a pLMB509 vector. Suicide vector-mediated homologous recombination was used to create variants as described previously (24), with 10 mM taurine in all media to ensure NDH-2 expression throughout. The point mutation cassette included homologous flanking regions 1,000 bp up- and downstream of *nqo13* (Pden_2232). A kanamycin (*Kan^R^*) selection marker followed the second flanking region. The same cassette was used to create the D648N^Nqo12^ variant as it also encompassed the C-terminus of Nqo12. The primers used to generate each mutant are given in Table S2 and correct mutagenesis confirmed by sequencing.

### Small-scale membrane preparations

*P. denitrificans* colonies were picked and grown for 24 h (30 °C, 225 rpm shaking) in 5 mL LB containing rifampicin (50 µg mL ^–1^), gentamicin (20 µg mL^–1^) and 10 mM taurine where required. The precultures were used to inoculate 50 mL LB (without antibiotics) in a 250 mL conical flask, incubated for 16 to 20 h (30 °C, 225  rpm) and harvested at late-log phase when OD _600_ was 3.0 to 4.5. Cells were collected using a Heraeus Primo centrifuge and resuspended in 1 mL resuspension buffer (50 mM MES pH 6.5 at 4 °C, 0.002% (w/v) phenylmethanesulfonyl fluoride (PMSF) and one complete EDTA-free protease inhibitor cocktail (Roche) per 50 mL). Then cells were lysed using a Q700 probe sonicator (QSonica) with a 1.6 mm microtip at 60% amplitude using a 5 s on/45 s off cycle for a total 1 min sonication. Membranes were collected by centrifugation (241,500 ×   *g*, MLA130 rotor, 1 h) and suspended in 50 mM MES pH 6.5 at 4 °C.

### Large-scale membrane preparations for purification

Membranes were prepared for the purification of complex I variants as described previously (24). During cell growth, NDH-2 expression was induced by the addition of 10 mM taurine for variants E141Q, K232Q, K263Q, E405Q, H320L, H246F, and L242A.

### Enzyme purifications

*P. denitrificans* complex I was purified from membranes as described previously (24). The purified complex was concentrated to 10 to 20 mg mL^–1^ before glycerol was added at 20% and the protein flash frozen in liquid N_2_. MaeB and FumC from *E. coli* (58) and AOX from *T. brucei brucei* (59) were purified as described previously.

### Preparation of SBPs and proteoliposomes

SBPs were prepared as described previously (38) but using a succinate minimal medium for cell growth rather than LB, and the cells were harvested at mid exponential phase (*OD*_600_ = ∼1.8 to 2.5). The media was adjusted to pH 7.2 and contained 50 mM succinate, 9.35 mM NH_4_Cl, 2 mM MgSO_4_, 0.07 mM CaCl_2_, 0.29 mM KH_2_PO_4_, 0.69 mM K_2_HPO_4_, 25.2 mM Na-HEPES, 19.6 μM Na_2_-EDTA, 9 μM FeSO_4_, 0.1 μM MnCl_2_, 0.8 μM CuCl_2_, 1 μM Na_2_MoO_4_, and 2.5 μM ZnCl_2_ (38, 60). Proteoliposomes were prepared and characterized as described previously (24) using a phospholipid composition of 80:10:10 (%, w/w) dioleoyl phosphocholine (DOPC): dioleoyl phosphoethanolamine (DOPE): tetraoleoyl cardiolipin (CDL).

### Kinetic assays

All kinetic assays were carried out at 32 °C in a Molecular Devices SpectraMax 348 96-well plate reader (Molecular Devices). Assays with membranes and soluble complex I were in buffer containing 10 mM MES (pH 6.5 at 32 °C), 25 mM NaCl, and 2 mM CaCl_2_. Assays with SBPs were in 10 mM Tris-SO_4_ (pH 7.5 at 32 °C) and 250 mM sucrose. Assays with proteoliposomes were in 10 mM  MES (pH 6.5 at 32 °C), 50 mM KCl and 250 mM sucrose, supplemented with 20 µg mL^–1^ AOX, and uncoupled with 0.5 µg mL^–1^ gramicidin A. NADH oxidation and NAD^+^ reduction were measured spectroscopically at 340 to 380 nm (*ε* = 4.81 mM^–1^ cm^–1^) and NADH:APAD^+^ (3-acetylpyridine adenine dinucleotide) oxidoreduction at 400 to 450 nm (*ε* = 3.16 mM^–1^ cm^–1^).

The complex I-specific substrate, deaminoNADH (dNADH) was used for all membrane and isolated complex I measurements in place of NADH. For NADH:O_2_ measurements, typically 10 µg mL^–1^ of membranes were assayed with 200 µM dNADH and 20 µg mL^–1^ alamethicin. Piericidin-insensitive background rates were measured by addition of 5 µM piericidin A. dNADH:AOX activity was measured by inhibiting complexes III and IV with 1 µM antimycin and 400 µM KCN, respectively, in the presence of 20 µg mL^–1^ AOX. dNADH:APAD^+^ activities of membranes were measured using 100 µM dNADH, 500 µM APAD^+^, and 1 µM piericidin A. Detergent-solubilized complex I measurements were typically performed with 1 µg mL^–1^ complex I in the presence of 0.15% asolectin and 0.15% CHAPS (3-[(3-cholamidopropyl)dimethylammonio]-1-propanesulfonate). dNADH:DQ activity was measured using 200 µM dNADH and 200 µM DQ and dNADH:APAD^+^ activity using 100 µM dNADH, 500 µM APAD^+^, and 1 µM piericidin A.

RET (succinate:NAD^+^ oxidoreduction) in SBPs was measured in SBP assay buffer supplemented with 2 mM MgSO_4_ and 1 mM K_2_SO_4_. RET measurements were initiated by addition of 5 mM NAD^+^ and 5 mM succinate to SBPs (20 µg mL^–1^). RET was normalized to complex I content in membranes as approximated by the dNADH:APAD^+^ activity of SBPs. dNADH:APAD^+^ was measured in SBP buffer containing 500 µM APAD^+^ and 100 µM dNADH.

### Determination of proton-pumping stoichiometries

ATP synthesis rates were compared to the rates of oxidoreduction from three different pathways, CI/CIII/CIV, CII/CIII/CIV, and CI/AOX, using a method based on previous established protocols (3, 40). Assays were performed at 32 °C, pH 7.5, and, to keep the conditions as consistent as possible, all the assays contained 20 µg mL^–1^ SBPs, 10 mM Tris-SO_4_, 250 mM sucrose, 10 mM KPO_4_, 2 mM MgSO_4_, 1 mM K_2_SO_4_, 1 mM ADP, 250 µM Ap5A (P^1^, P^5^-di(adenosine-5′) pentaphosphate to inhibit adenylate kinase), 20 units mL^–1^ SOD, 5000 units mL^–1^ catalase, 5 mM succinate, and 20 µg mL^–1^ AOX. For the CI/CIII/CIV pathway, complex II was inhibited with 2 µM atpenin A and AOX with 1 µM ascofuranone and 200 µM dNADH was added to initiate catalysis. For the CII/CIII/CIV pathway, complex I was inhibited with 2 µM piericidin A and AOX with 1 µM ascofuranone and the buffer also contained 2 mM NADP^+^, 240 µg mL^–1^ FumC and 1.2 mg mL^–1^ MaeB to detect succinate oxidation as NADP^+^ reduction (58). For the CI/AOX, pathway, complex II was inhibited with 2 µM atpenin A and complexes III and IV with 1 µM antimycin A and 400 µM KCN, respectively, and catalysis was initiated by 200 µM dNADH. Finally, to modulate the substrate oxidation rates to match the ranges of ATP synthesis activities, piericidin A and atpenin A were titrated for the respective CI- and CII-driven pathways. For each datapoint, substrate oxidation rates were monitored spectroscopically in real time, and ATP synthesis was measured over a 3 min time course. Starting after 1 min, 10 μL aliquots of reaction mixture were withdrawn and quenched into 40 μL of 4% (v/v) trifluoroacetic acid, followed 20 s later by addition of 250 µL neutralizing buffer (1 M Tris-SO_4_ pH 8.0). Each quenched sample was then pipetted (three 80 µL aliquots) into a 96-well, white, flat-bottom luminescence plate, and 20 µL of the Roche ATP Bioluminescence Assay Kit CLS-II kit reagent was added to each well. The luminescence (580 ± 80 nm) of each well was measured using a BMG Labtech CLARIOstar plus microplate reader fitted with a luminescence aperture, and the ATP concentrations calculated by comparison to a standard curve generated alongside from samples of known ATP concentrations.

The proton-pumping stoichiometry (*n*^CI^) for each variant was calculated using the equations developed by Jones et al. (2017) (3), considered under conditions where the rates of ATP synthesis depends linearly on the rates of substrate oxidation to enable the gradients of linear plots ($gradient,\ \mathit{ m}\ = \ \Delta {\mathit{ v}}_{{\rm{ATP\ synthesis}}}/\Delta {\mathit{ v}}_{{\rm{substrate\ oxidation}}}$) to be compared as described by Fedor and Hirst (2018) (40). Using the well-established proton-pumping stoichiometry of 6 for CIII + CIV catalysis (42, 43)

(1) $$\begin{equation*}
{\rm{\ }}{n}^{{\rm{CI}}} = \frac{6}{{\left( {\frac{{{m}_{{\rm{CI}}:{\rm{CIII}}:{\rm{CIV}}}}}{{{m}_{{\rm{CI}}:{\rm{AOX}}}}} - 1} \right)}},\
\end{equation*}
$$

(2) $$\begin{equation*}
{\rm{\ }}{n}^{{\rm{CI}}} = \ 6 \times \left( {\frac{{{m}_{{\rm{CI}}:{\rm{CIII}}:{\rm{CIV}}}}}{{{m}_{{\rm{CII}}:{\rm{CIII}}:{\rm{CIV}}}}} - 1} \right),
\end{equation*}
$$

(3) $$\begin{equation*}
{\rm{\ }}{n}^{{\rm{CI}}} = \ 6 \times \left( {\frac{{{m}_{{\rm{CI}}:{\rm{AOX}}}}}{{{m}_{{\rm{CII}}:{\rm{CIII}}:{\rm{CIV}}}}}} \right).
\end{equation*}
$$

First, pairwise comparisons of the gradients were used to determine three values for *n*^CI^ directly from Equation 1 to 3 (Figure 4B). Second, by setting a test value of *n*^CI^ the three gradients can all be reduced to a function of a single variable gradient, and the error of the fit then minimized by varying this single gradient value (Figure 4C and Figure S5).

### SDS-PAGE analyses

Purified complex I was incubated in loading buffer (0.125 M Tris-HCl (pH 6.8), 20% (w/v) glycerol, 4% (w/v) SDS, 0.005% (w/v) bromophenol blue and 0.1 M DTT) for 10 min at room temperature and 10 µg loaded on a Novex WedgeWell 10% to 20% tris-glycine gel. The gel was run as described previously and bands visualized using Coomassie R250 (61).

### NanoDSF

Nano differential scanning fluorimetry (nanoDSF) was performed in a Prometheus NT.48 (NanoTemper Technologies). Isolated complex I was diluted to 0.3 mg mL^–1^ in buffer containing 20 mM MES pH 6.5 at 4 °C, 150 mM NaCl, 10 mM CaCl_2_, 10% (v/v) glycerol, 0.05% (w/v) DDM and loaded in triplicate into 10 µL capillaries. The fluorescence at 330 and 350 nm was recorded as the temperature was increased from 20 to 80 °C at a rate of 4.5 °C min^–1^ with an excitation wavelength of 280 nm and the excitation power between 57% and 65%.

## Supplementary Material

pgac276_Supplemental_File

## Data Availability

All data generated or analyzed during this study are included in this published article (and its Supplementary Material files).
