# Supplementary material for: Membrane-domain mutations in respiratory complex I impede catalysis but do not uncouple proton pumping from ubiquinone reduction
Source: PNAS Nexus. 2022 Dec 2;1(5):pgac276. doi: 10.1093/pnasnexus/pgac276 (PMC9802314; doi:10.1093/pnasnexus/pgac276)
Supplement: pgac276_Supplemental_File [file pgac276_supplemental_file.docx]

**Supplementary Information**

**Membrane-domain mutations in respiratory complex I impede catalysis but do not uncouple proton pumping from ubiquinone reduction**

Owen D. Jarman and Judy Hirst^*^

The Medical Research Council Mitochondrial Biology Unit, University of Cambridge, The Keith Peters Building, Cambridge Biomedical Campus, Hills Road, Cambridge CB2 0XY, UK

**^*^**Corresponding author

**Table S1.** The numbering for equivalent residues mutated in this study in other key model organisms. Residues in italics are not conserved. The amino acid substituted for each residue is shown.

| **Set** | ***Paracoccus denitrificans*** | ***Bos taurus*** | ***Escherichia coli*** | ***Yarrowia lipolytica*** | ***Thermus thermophilus*** | **TMH location** | **Mutation** | **Comment** |
| --- | --- | --- | --- | --- | --- | --- | --- | --- |
| **Set 1** | E141 | E123 | E144 | E142 | E123 | TMH5 | Q | Ion-pair residue with K232 in central axis |
|  | K232 | K206 | K234 | K221 | K204 | TMH7 | Q | Ion-pair residue with E141 in central axis |
|  | K263 | K237 | K265 | K252 | K235 | TMH8 | Q | Proton transfer site in central axis |
|  | E405 | E378 | E407 | E395 | E377 | TMH12 | Q | Proton transfer exit site in central axis |
|  | H320 | H293 | H322 | H309 | H292 | TMH10 | L | Proton transfer site in central axis |
|  | H346 | H319 | H348 | H335 | *S318* | TMH11 | Q | Proton transfer site in central axis |
| **Set 2** | D648^Nqo12^ | D554 | D563 | D580 | D557 | - | N | Coordination of the transverse helix to Nqo13 |
|  | Y315 | Y288 | Y317 | Y304 | Y287 | TMH10 | F | Coordination to D648N on transverse helix |
|  | H239 | H213 | H241 | H228 | H211 | TMH7 | Q | Coordination to D648N on transverse helix |
| **Set 3** | L242 | L216 | L244 | L231 | L214 | TMH7 | A | Potential Leu gate residue |
|  | H246 | H220 | H248 | H235 | H218 | TMH7 | F | Proton uptake |
|  | A314 | A287 | A316 | A303 | A286 | TMH10 | L | Near to L242 |
|  | W241 | W215 | W243 | W230 | W213 | TMH7 | F | Involved in potential Leu gate mechanism |
|  | R367 | R340 | R369 | R357 | *L339* | - | H | Site of clinical mutation that causes LHON |

**Table S2.** Primers used to generate the complex I variants. The base pairs that have been altered from the wild-type sequence are highlighted in red. The reverse (Rev) strand and the forward (Fwd) strand primers are shown.

| Mutation | Primer (5ʹ–3ʹ) | Original codon | Variant codon |
| --- | --- | --- | --- |
| E141Q Fwd | GGAAGAACAGGTAGAACAGCACCAG | GAG | CAG |
| E141Q Rev | AGGCGGGGCTGATCCCGATG |  |  |
| K232Q Fwd | GGACCGCGAAGCTGGCAAAG | AAG | CAG |
| K232Q Rev | AGATGCCGATGTGGCCGGTC |  |  |
| K263Q Fwd | GCAGCAGCACCGCGGCCAGCAG | AAG | CAG |
| K263Q Rev | AGATGGGCGGCTACGGCTTC |  |  |
| E405Q Fwd | GGCCCACGAAACCCGAGGTG | GAG | CAG |
| E405Q Rev | AGTTCCTGACGCTGATGGGG |  |  |
| H320L Fwd | CGTAGCCCATCAGGGCGACCGAG | CAT | CTG |
| H320L Rev | TGACCATGGGCGTCTTTGCC |  |  |
| H346Q Fwd | GAGATAAAGCCCTGCGACAGCATC | CAC | CAG |
| H346Q Rev | GGGCGCGCTGTTCCTGTGCG |  |  |
| D648N^Nqo12^ Fwd | TGCCGCCCTTCCACAGCACG | GAC | AAC |
| D648N^Nqo13^ Rev | ACGGCGCCGTCATCGACGGC |  |  |
| Y315F Fwd | GAAGGCGATGACCTTCTTCATG | TAT | TTC |
| Y315F Rev | TCCTCGGTCGCCCATATGGG |  |  |
| H239Q Fwd | CTGGACCGGCCACATCGGC | CAT | CAG |
| H239Q Rev | ACCTGGCTGCCCGACGCGC |  |  |
| L242A Fwd | GCGCGTCGGGCGCCCAGGTATGG | CTG | GCG |
| L242A Rev | ACGTCCAGGCGCCGACCGCC |  |  |
| H246F Fwd | GCGCCTGGACGAACGCGTCGGGC | CAC | TTC |
| H246F Rev | CGACCGCCGGCTCGGTCCTG |  |  |
| W241F Fwd | CGTCGGGCAGGAAGGTATGGACC | TGG | TTC |
| W241F Rev | CGCACGTCCAGGCGCCGACC |  |  |
| A314L Fwd | CCGAGGAATACAGGATGACCTTC | GCC | CTG |
| A314L Rev | TCGCCCATATGGGCTACGTG |  |  |
| R367H Fwd | CGTCGATCTCGTGCGTGTGCATG | CGC | CAC |
| R367H Rev | CCTATGGCGGGTTGGTGAAC |  |  |

**Figure S1.** Elution profile of wild type and variants analyzed by size-exclusion chromatography. Complex I from each variant was first isolated and the purified samples were subsequently diluted in gel filtration buffer (20 mM MES pH 6.5 at 4 ˚C, 150 mM NaCl, 10 mM CaCl_2_, 10% glycerol, 0.05% DDM) and loaded onto a Superdex 200 increase 5/150 GL size-exclusion column (~200 µg of complex I) at a flow rate of 0.15 mL min^–1^. The absorbance at 280 nm is shown for each sample and for comparison, the wild-type elution profile is shown as blue dashed lines overlayed onto each variant shown in red.

**Figure S2.** NanoDSF fluorescence traces for wild-type and variant complex I. Each isolated complex I variant was diluted to 0.3 mg mL^–1^ and loaded into a capillary in triplicate (10 µL). The fluorescence at 330 nm and 350 nm was recorded in a Prometheus NT.48 (NanoTemper Technologies) as the temperature was increased from 20 ˚C to 80 ˚C at a rate of 4.5 ˚C min^–1^. The first derivative of the 330/350 nm ratio is shown for each variant in red and is overlayed with the wild type in blue. The trace shown is the average reading for three samples.


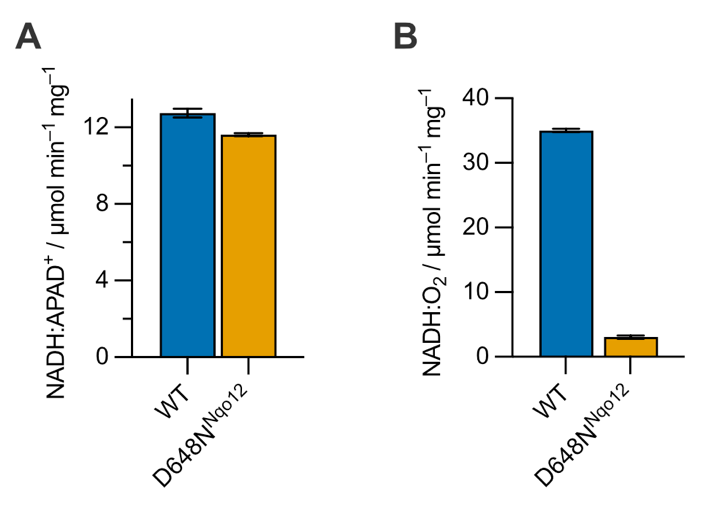


**Figure S3.** Catalytic activities for wild-type and D648N^Nqo12^ complex I reconstituted into proteoliposomes. The activities are given per mg of outward-facing complex I and presented as the average of three technical replicates ± S.E.M. All activities were measured in buffer containing 10 mM MES pH 6.5 at 32 °C, 50 mM KCl and 250 mM sucrose. (**A**) NADH:APAD^+^ activities for the reconstituted wild-type and D648N^Nqo12^ enzymes. (**B**) NADH:O_2_ activities for the reconstituted wild-type and D648N^Nqo12^ enzymes. Proteoliposomes were prepared as described previously^24^ using a lipid composition of (80:10:10 dioleoyl phosphocholine (DOPC):dioleoyl phosphoethanolamine (DOPE):tetraoleoyl cardiolipin (CDL) (%, w/w)), supplemented with 20 µg mL^–1^ AOX and uncoupled with 0.5 µg mL^–1^ gramicidin A.


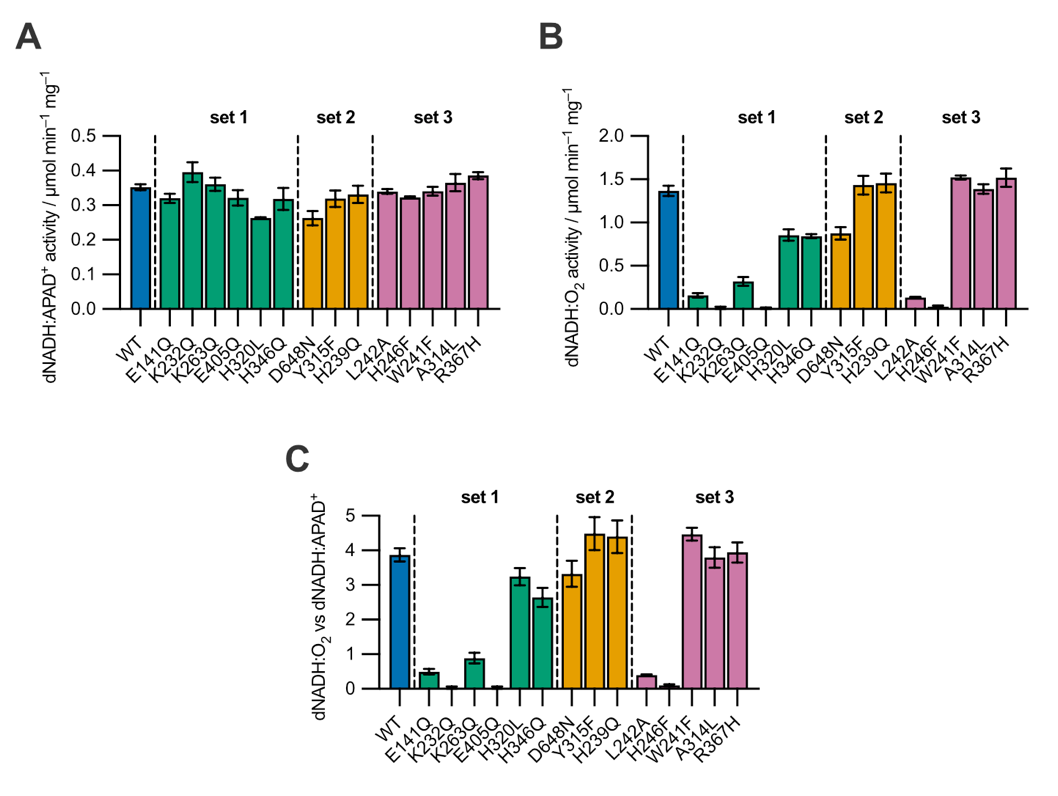


**Figure S4.** Activities of the wild-type and variant membranes used for the preparation of purified complex I samples. Membranes were prepared in large quantities as described in the Materials and Methods. (**A**) dNADH:APAD^+^ activities; (**B**) dNADH:O_2_ activities; (**C**) dNADH:O_2_ activities normalized to dNADH:APAD^+^ rates (to account for variations in complex I content between membranes preparations). The data shown are the averages of three technical replicates ± S.E.M.


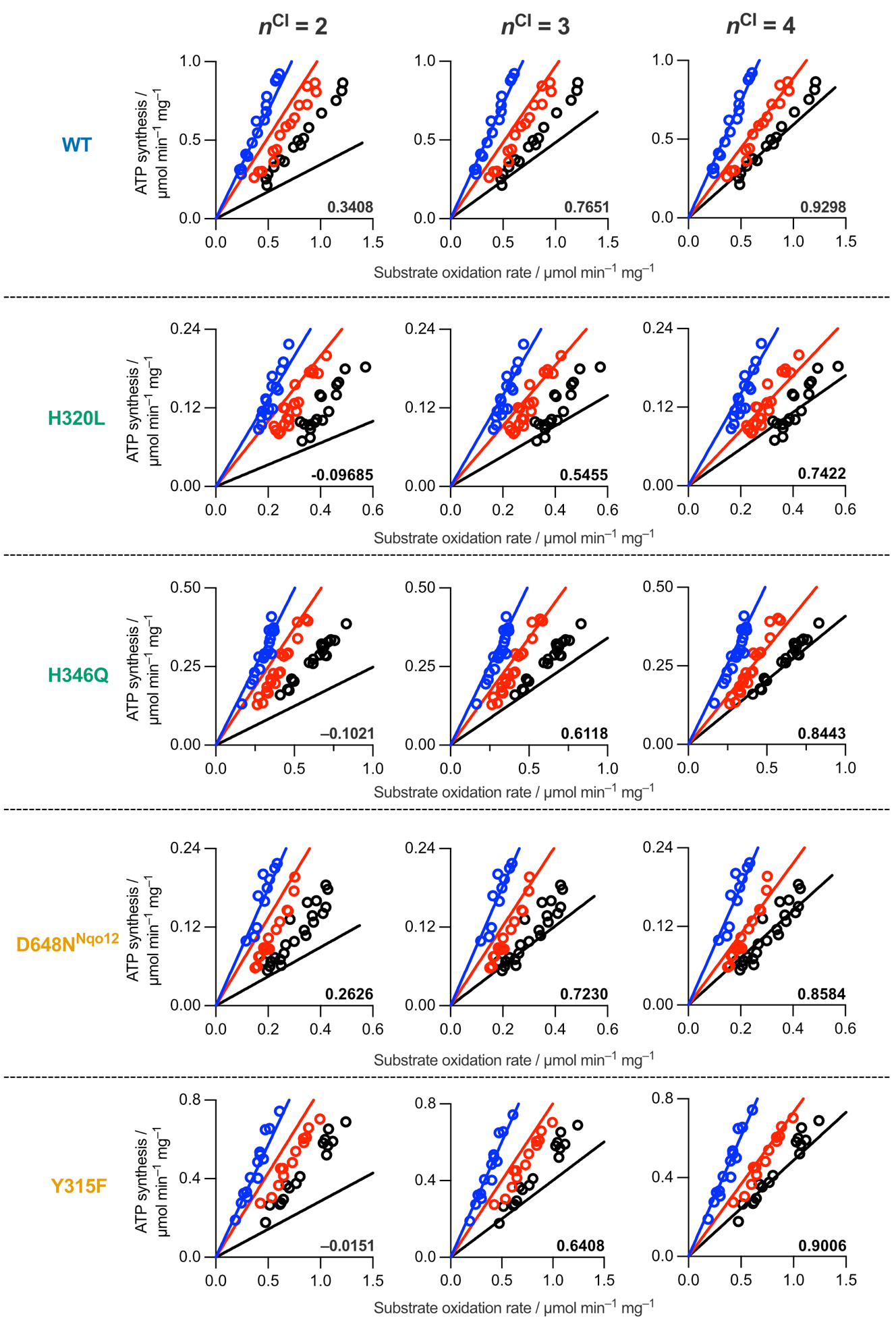


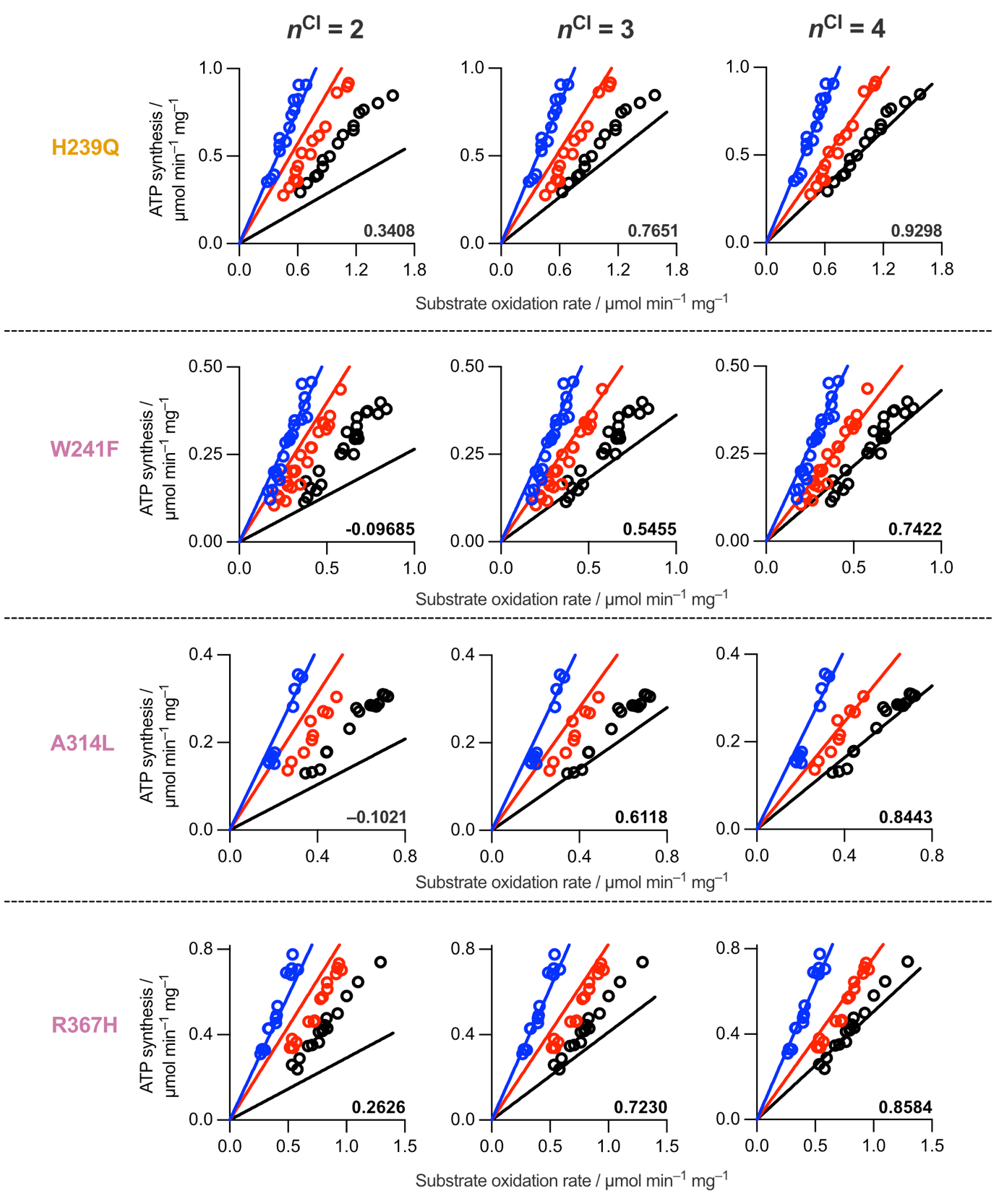


**Figure S5.** Simulated global fits for the proton-pumping stoichiometry data given in **Figure 4** for *n*^CI^ = 2, 3 and 4. Experimental data points and the fits for CI/CIII/CIV (blue), CII/CIII/CIV (red) and CI/AOX (black) are shown. The *R*^2^ values (where *R*^2^ is the ‘goodness of fit’ between the data and the model) are given on the graphs. In all cases, the *R*^2^ values are highest when *n*^CI^ = 4.

**
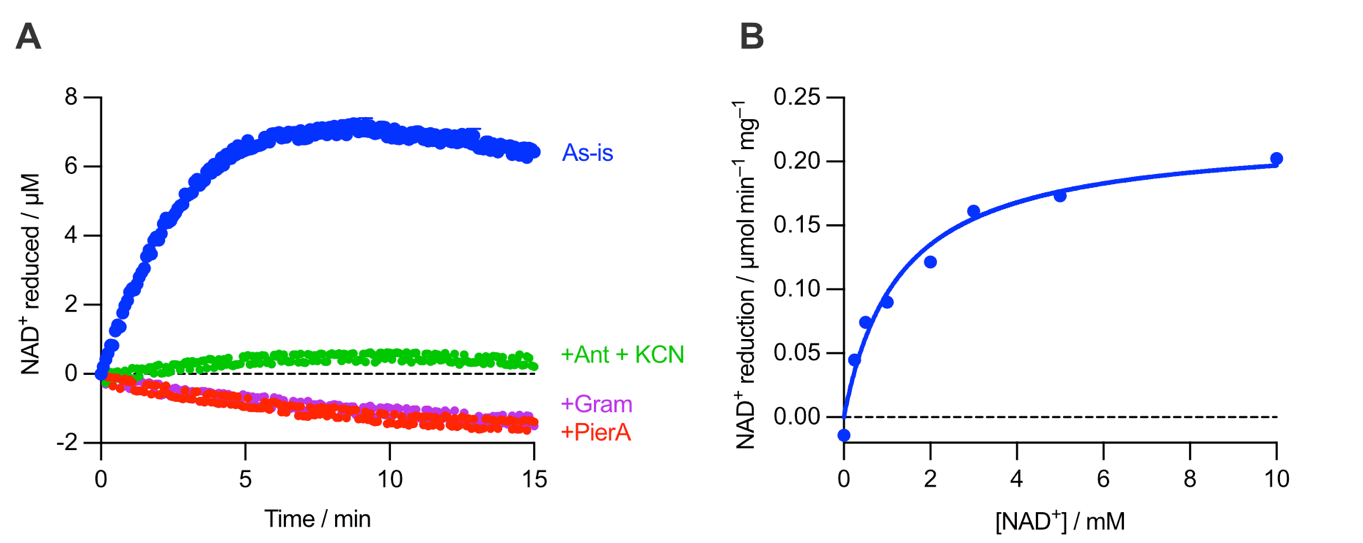
**

**Figure S6.** Measuring RET using wild-type *P. denitrificans* SBPs. (**A**) Demonstration of RET in SBPs showing the total concentration of NAD^+^ reduced as a function time. The rate of RET was determined from the initial slope, when the rate is linear (blue). RET was abolished by the membrane uncoupler gramicidin A (150 ng mL^–1^) (purple), by piericidin A (2 µM) to inhibit complex I (red) and when complexes III and IV were inhibited with 1 µM antimycin and 400 µM KCN, respectively (green). (**B**) *K*_M_ curve showing the dependence of the rate of RET on NAD^+^ concentration using 20 µg mL^–1^ SBPs. The *K*_M_ value is 1.30 ± 0.13 mM.
